# Gynæcology in General Practice

**Published:** 1897-11-20

**Authors:** 


### GYNÆCOLOGY IN GENERAL PRACTICE.

There is a certain type of case which has been and still is the foundation and source of the great mass of the ordinary daily work of the gynæcologist; a type of case which on the one hand is largely preventable by the exercise of care, and on the other hand is capable of very efficient treatment by a well-equipped general practitioner. Clearly, then, it is of great importance to all practitioners to recognise these cases. Dr. Berry Hart, writing in the *Practitioner*, says that the vast majority in minor cases in out-patient gynæcological clinics make in many respects the same complaint. They say that since their last labour, or since some abortion, they have not felt well; that they have occasional pains in the back—over the sacrum—at the side, or below the left breast; that between their periods they have an excess of white discharge, while at the period the flow, as a role, is increased. Now, if such a case be examined by the bimanual method, it is found that the lesion is multiple; and it is very important that this should be recognised, for no doubt the multiple nature of the lesion is the reason why these cases have been for so long the battlefield of treatments, one man concentrating his attention upon one aspect, and another on another, of what really is a multiple affair. The cervix uteri is more or less lacerated, or, at any rate, the os externum is patulous; the uterus may be retroflexed—in fact, it usually is; the uterus is generally a little enlarged, and there may be some inflammatory thickening in its neighbourhood, either in the peritoneum, cellular tissue, ovary, or tube. But the case is not one of mere disease of the cervix, nor of mere retroflexion, nor of either peri- or parametritis alone; it is a combined condition, in which the various lesions concerned have arisen as a result of septic absorption from the endometrium or cervix. The great clinical cause of such cases is neglected abortion, and cases of labour in which there has been difficulty in the third stage, and the final condition is one of endometritis with secondary thickenings. The treatment of this condition may be given in few words; may, in fact, be put in the form of three simple rules: (1) When there is menorrhagia and leucorrhœa single or combined, with inflammatory thickenings, but without suppuration, curette the uterus to begin with. (2) Where the menorrhagia and leucorrhœa are not marked, but the main condition is one of minor inflammatory thickening, the cotton wool tampon and hot douche may be used. The wool should not be "absorbent," and it may be used either with ichthyol and glycerine (1 to 20) or with boric acid in fine powder sprinkled over vaseline. This may be inserted by the patient in the morning (after a little practice) and removed at night, when a hot douche may be used. (3) The retroflexion, if not complicated by some prolapse of the pelvic floor, is probably best left alone.

There will always be work for the specialist, for the more careful the general practitioner is the more strange cases does he discover, many of which he will find it desirable to hand over to those who have had special experience. Nevertheless a considerable portion of the work now done by the specialist might well fall to the family medical man, and Dr. Berry Hart has done well in pointing out how large is the group which he describes as the "chronic infected case," and how fairly amenable it is to a line of treatment which is well within the reach of the well-equipped practitioner.

